# Effects of biogas slurry fertilization on fruit economic traits and soil nutrients of *Camellia oleifera* Abel

**DOI:** 10.1371/journal.pone.0208289

**Published:** 2019-05-09

**Authors:** Lu You, Shuqin Yu, Huiyun Liu, Chutian Wang, Zengliang Zhou, Ling Zhang, Dongnan Hu

**Affiliations:** 1 College of Forestry, Jiangxi Agricultural University, Nanchang, Jiangxi, China; 2 Jiangxi Provincial Key Laboratory of Silviculture, Jiangxi Agricultural University, Nanchang, China; Pennsylvania State University, UNITED STATES

## Abstract

*Camellia oleifera* Abel (C. *oleifera*) absorb nutrients from surrounding soils and its yield is highly influenced by these nutrients and by fertilizer application. Thus, the soil nutrients play a central role in C. *oleifera* production. This study investigated the effects of biogas slurry applications on soil nutrients and economic traits of *C*. *oleifera* fruits. Five different amounts of biogas slurry (0, 10, 20, 30, or 40 kg/plant/year, three applications per year) were used as fertilizer for *C*. *oleifera* plants in 2015 and 2016. The nutrients of rhizosphere soil and the economic traits, including fruit yield, seed rate, and oil yield of *C*. *oleifera* fruit, were measured each year. The results showed that fertilization with biogas slurry significantly increased soil organic matter, available nitrogen (N), phosphorus (P), and potassium (K) both in 2015 and 2016. Increases in soil available N, P, and K were maximal in the highest slurry application group followed by the second highest application group. The oil yield correlated with the content of soil available P in both 2015 and 2016, and with soil organic matter in 2015. Fertilization with biogas slurry decreased the saturated fatty acid content in fruit but had no effect on the unsaturated fatty acid content. In conclusion, fertilization with biogas slurry increased rhizosphere soil nutrients and fruit economic traits of *C*. *oleifera* and rates of at least30 kg/plant/year had the most positive effects. This study expands the knowledge of fertilization with biogas slurry in C. *oleifera* production.

## Introduction

Biogas slurry is a secondary product produced by anaerobic fermentation of bio-materials, which has been widely used as a fertilizer in agricultural production. Biogas slurry is not only an environmentally friendly organic fertilizer, but also an efficiently utilized waste material. Recently, livestock excrements, such as feces and urine, have become a severe problem in China. These challenge many animal premises and create extensive environmental pollution [1]. Anaerobic fermentation is one of the most effective solutions for this challenge. The main product of anaerobic fermentation is biogas, which is an important and clean energy. The by-product, biogas slurry can be used in agricultural and forestry production [2, 3]. Currently, the use of biogas slurry as a fertilizer has drastically increased in China and many other Asian countries, not only due to the considerable cost of chemical fertilizers, but also to utilize the high nutrient level in biogas slurry [4, 5]. It has been reported that more than 450 million tons of biogas slurry are being used in China each year [6]. Biogas slurry has two main uses for plant production: it is used as a bio fertilizer with high levels of nitrogen (N), phosphorus (P), potassium (K), and other trace elements, and it is as a biological pesticide due to its high levels of amino acids, growth hormones, and antibiotics, all of which promote plant growth [7, 8]. It has been reported that biogas slurry contains abundant nitrogen, which is a readily available nutrient. After fermentation, the content of ammonium ions (NH_4_^+^) and pH of the biogas slurry increased, while the concentration of carbon (C) from the dry matter decreased, and the C/N ratio also decreased [9, 10]. Furthermore, biogas slurry supplies more plant-readily available N than other fertilizers [11].The available nitrogen can be directly absorbed by plants, including inorganic nitrate (NO_3_) and ammonium (NH_4_) and as simple structured organic partly from the degradation of organic matter.

*Camellia oleifera* Abel (*C*. *oleifera*) is an oil tree species that is native to China with a distribution in 18 provinces/cities and *C*. *oleifera* is cultivated in more than 1,000 districts in China. It has been reported that the planting area of *C*. *oleifera* in China exceeds 65 million acres [12]. Camellia oil, the product of *C*. *oleifera*, is a high quality edible oil that is characterized by abundant unsaturated fatty acids, including oleic acid and linoleic acid [13, 14]. China has a long tradition of cooking with Camellia oil, especially in South China. In recent years, the area planted with *C*. *oleifera* is expanding since the demand for oil is increasing [15]. One of the key factors that determine the yield of *C*. *oleifera* is fertilization [16]. Traditional cultivation methods mainly depend on chemical fertilization, farm insecticides, and chemical growth hormones, all of which could lead to acidification and hardening of soil, nutrient imbalance, and regression, which ultimately results in production recession [17–19].

Positive effects of biogas slurry and other organic material on plants and crops have been documented [20]. Liquid fermented biogas slurry, from the outlet of the biogas digester, can be readily used and directly applied to crops, vegetables, fodder grass, and many other plants [21–23]. However, specific knowledge about the effects of biogas slurry on the production of *C*. *oleifera* and the soil nutrients remains limited. The potential benefits of biogas slurry for *C*. *oleifera* and its application at different amounts need to be elucidated. This study investigated the effects of biogas slurry applications on the soil nutrients, the fruit yield, and fruit quality of *C*. *oleifera* to assess whether biogas slurry could partly or wholly substitute chemical fertilizers.

## Materials and methods

### Ethics statement

This study was conducted in a private *C*. *oleifera* plantation in Wannian, Jiangxi province in China from 2015 to 2016. The owner of the land and plantation had given permission to conduct the study on this site.

### Materials

The investigated area has a typical warm and humid subtropical monsoon climate with an annual mean temperature of 17°C, an annual rainfall level of 1808 mm, and an annual relative humidity of 82%. The annual number of mean frost-free days is 259 d in the experimental area. *C*. *oleifera* trees were planted in red clay soil on sunny and hilly land with a gradient of less than 20%. Ganwu strains were used in this study, the plantation was seven years old, the row spacing was 3 by 3 m, and trees had a height of 2–3 m. The biogas slurry was fermented from pig farm yard manure, using a farm biogas digester with a 200 m^3^ capacity for 30 days. The average characteristics of biogas slurry were detected, as: pH = 8.040 ± 0.020, total N = 0.680 ± 0.032 g/kg, total P = 0.086 ± 0.007g/kg, total K = 3.620 ± 0.041 g/kg, ammonium = 0.522 ± 0.066 g/kg, percent of organic matter = 0.042 ± 0.003%.

### Experimental design

The experiment was conducted using a randomized block design with five treatments according to the level of applied biogas slurry: (1) no biogas slurry [group B_0_]; (2) 10 kg of biogas slurry/plant/year [group B_1_]; (3) 20 kg of biogas slurry/plant/year [group B_2_]; (4) 30 kg of biogas slurry/plant/year [group B_3_]; (5) 40 kg of biogas slurry/plant/year [group B_4_]. All five treatments did not receive fertilization with chemical fertilizers. The biogas slurry was fertilized three times a year (March, June, and September) with the furrow method into the drip line of trees (Table 1). The biogas slurry was weighed according to the required amount for each treatment, and mixed with the same weight of clean water and applied to each plot. Each treatment was conducted with three blocks with five replicate plants per plot.

**Table 1:** Experiment design (Unit: Kg/plant).

Treatments	March	June	September	Annual total
B_0_	0	0	0	0
B_1_	4	3	3	10
B_2_	8	6	6	20
B_3_	10	10	10	30
B_4_	14	13	13	40

### Soil collection and physical-chemical analyses

Immediately after fruit harvest, mixed soil samples were collected from five replication plants in each plot. These soil samples were cleared of roots and all other organic debris and subsequently air-dried, ground, and sieved (1 mm) for analysis of soil available nutrition and further sieved (0.149 mm) for organic matter detection. Organic matter was estimated via organic carbon using the conventional conversion: organic matter = 1.724 × organic carbon; organic carbon was determined by the Walkley-Black wet oxidation method; available N was estimated with the Kjeldahl method; available P was extracted with 1 M NH_4_F and 0.5 M HCL and estimated via the molybdenum-antimony colorimetric method; available K was extracted with neutral 1M NH_4_OAC and was estimated by flame emission spectroscopy [24].

### Fruit collection and analysis

Fruits were harvested in October and single tree yield was calculated by immediately weighing all fresh fruit from each tree. Thirty representative fruit samples of each tree were collected. Because *C*. *oleifera* yield fluctuates each year [25, 26], the production trait indices were calculated by using the average statistics of two years (2015 and 2016). After seeds were dried at 80°C to constant mass, these were weighted and powdered by a high speed disintegrator with high rotation (TW100, Taisite, China). About 1.0000 g of ground sample was weighed as w_0_ (g), then transferred to Soxhlet extraction using petroleum ether (60–90°C) at 80°C for 12h. After the solvent was evaporated in vacuum, the residual was dried at 60°Cto a constant weight of w_1_ (g) in vacuum. The oil weight was calculated according to the formula: oil weight = w0—w_1_. Fatty acids in fresh fruits were measured according to the Chinese Standard (GB-5009, 168–2016 method), by a gas chromatograph (GC-2010 Plus, Shimadzu, Japan) [27–29]. The following fruit characteristics were evaluated as follows: moisture rate of fresh seed = (fresh seed weight—dry seed weight) / fresh seed weight × 100%, fresh seed rate = (fresh seed weight / fresh fruit weight) × 100%, dry seed rate = (dry seed weight / fresh fruit weight) × 100%, oil rate of kernel = (oil weight / kernel weight) × 100%, oil rate of fresh fruit = oil rate of kernel × dry seed rate × 100%.

### Statistical analysis

The concentrations of organic matter, available N, P, and K between 2015 and 2016 were statistically analyzed by ANOVA using SPSS 19.0. Means were compared by least significant difference (LSD) tests at p < 0.05, and the data in the result represent the average ± STD. The effects of biogas slurry on soil nutrients (organic matter, available N, P, and K) and yield were tested via correlation analysis.

## Results

### Effects of biogas slurry on organic matter in rhizosphere soil

**Fig 1:**
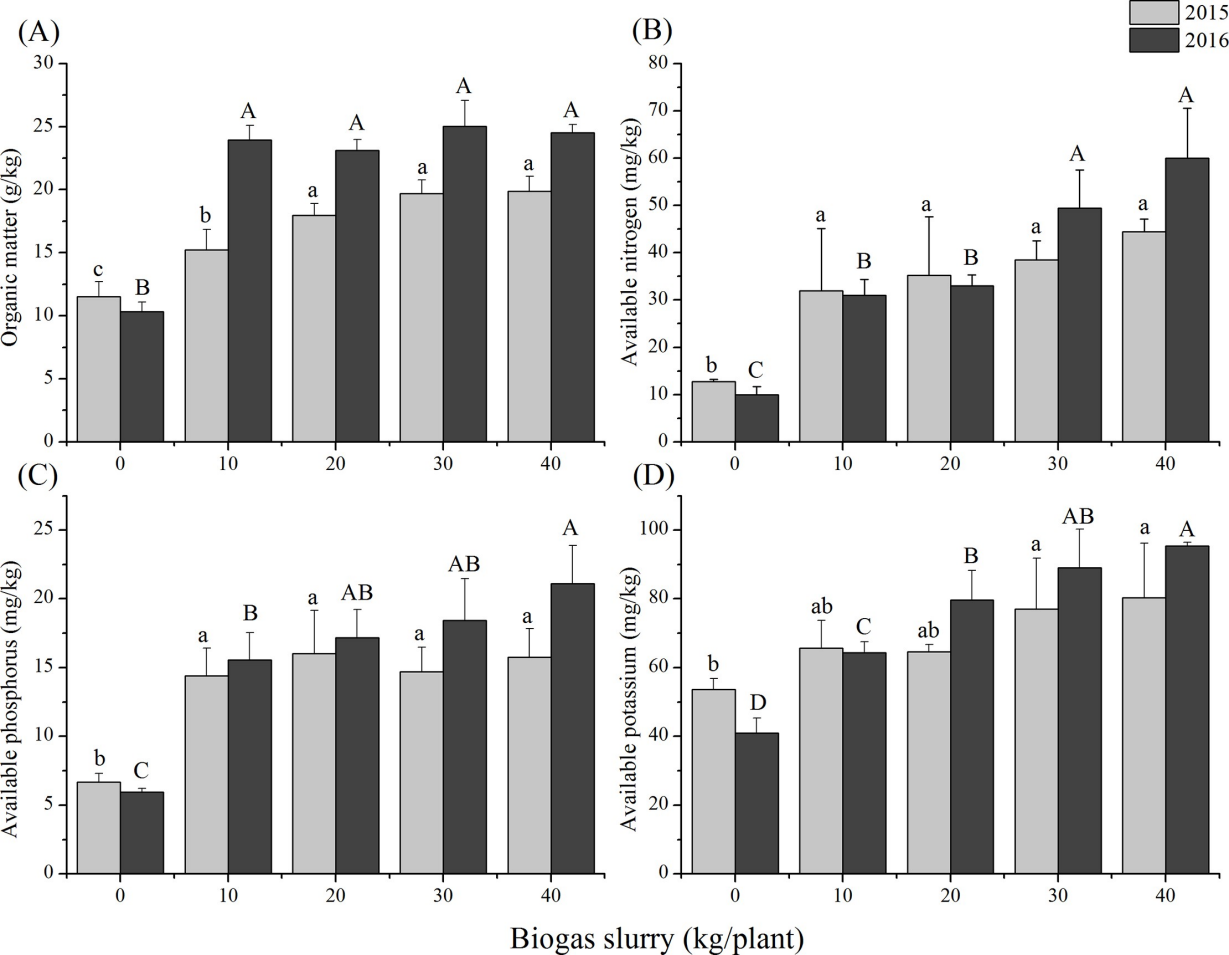
Effects of biogas slurry on contents of (A) organic matter, (B) available nitrogen, (C) phosphorus and (D) potassium in rhizosphere soil during 2015 and 2016. Different small and capital letters indicate the significant differences among five levels of biogas slurry addition in 2015 and 2016, respectively (p<0.05).

Fertilization with biogas slurry significantly increased the organic matter concentration of rhizosphere soils (Fig 1A and S1 Table). During the first experimental year (2015), concentrations of soil organic matter increased with increasing dose of biogas slurry. Compared to the control group B_0_, the organic matter concentration of B_1_, B_2_, B_3_, and B_4_ groups increased by 32.2% (p<0.05), 55.8% (p<0.01), 70.9% (p<0.01), and 72.6% (p<0.01), respectively. Multiple comparisons showed significant increases of soil organic matter between the fertilized groups and control group; no significant differences were found among treatments B_2_, B_3_, and B_4_. During the second experimental year (2016), the situation was similar to that in 2015. All biogas slurry application rates led to increased organic matter compared the control group. Treatments B_3_ and B_4_ achieved higher enhancement rates than those in 2015, with increments of 142.28% and 137.56%, respectively.

### Effects of biogas slurry on available nitrogen in rhizosphere soil

During the experiment, soil available nitrogen decreased from 2015 to 2016 in the control group. Fertilization with biogas slurry increased soil available nitrogen both in 2015 (p = 0.009) and 2016 (p = 0.000) (Fig 1B and S1 Table). When compared to the control group, treatment B_4_, (the treatment with the most biogas slurry fertilization), resulted in the highest improvement of available nitrogen in both 2015 (249.07%) and 2016 (499.2%). Low slurry application (B_1_, and B_2_) led to similar levels of soil available N in the two experimental years; however, at the second highest slurry addition rate (B_3_), soil available nitrogen continued to increase in 2016.

### Effects of biogas slurry on available phosphorus in rhizosphere soil

All four biogas slurry fertilized groups had higher concentrations of available phosphorus in 2016 than in 2015 (Fig 1C and S1 Table). However, the control group had lower available phosphorus concentrations in 2016 than in 2015. In 2015, the four fertilized groups (B_1_, B_2_, B_3_, and B_4_) had increments of 151.81%, 139.97%, 119.96%, and 135.82% of available P, respectively, compared to the control group. Multiple comparisons showed no significant differences among the four fertilized groups in 2015. In 2016, compared to the control group, the enhancement of available P was larger with higher slurry addition rates (B_1_, B_2_, B_3_, and B_4_) with increments of 161.95%, 188.88%, 210.10%, and 255.05%, respectively.

### Effects of biogas slurry on available potassium in rhizosphere soil

Available K decreased from 2015 to 2016 in both the control and the lowest slurry addition (B_1_) treatments (Fig 1D and S1 Table). In 2015, available K in soils increased with increasing amount of biogas slurry application, especially for treatments B_3_ and B_4_ (43.46% and 49.68%, respectively). In 2016, treatments B_3_ and B_4_ still showed significant enhancements of 117.07% (p < 0.01) and 132.52% (p < 0.01), respectively.

### Effects of biogas slurry on fruit yield and main economic traits of *C*. *oleifera*

The average of fruit yield and oil yield of *C*. *oleifera* in both 2015 and 2016 showed highest enhancement when at least 30 kg biogas slurry per plant each year was used (Table 2 and S2 Table). A growth trend in oil yield was found in response to increasing biogas slurry application. Regarding the main economic traits of *C*. *oleifera*, the fresh seeds from treatments B_0_ and B_1_ contained the highest moisture ratios, and the lowest oil yield. Compared to the control treatment B_0_, the fruit yield of B_3_ and B_4_ increased by 40.1% and 16.24%, respectively, and the oil yield increased by 105% and 95%, respectively.

**Table 2:** Effects of biogas slurry on yield and the main properties of *C*. *oleifera*.

Treatments	Yield kg/plant	Moisture rate of fresh seed (%)	Fresh seed Rate (%)	Dry seed Rate (%)	Oil rate of Kernel (%)	Oil rate of fresh fruit (%)	Oil yield kg/plant
B_0_	1.97±0.60^a^	44.68±2.03^a^	54.39±8.17^b^	33.84±7.98^a^	45.24±3.50^b^	10.52±3.14^b^	0.20±0.02^b^
B_1_	2.20±1.15^a^	44.36±2.66^a^	59.23±14.07^ab^	33.17±9.13^a^	49.28±3.16^b^	10.31±3.92^b^	0.23±0.12^b^
B_2_	2.24±0.91^a^	40.49±0.11^b^	66.82±15.98^ab^	39.77±9.54^a^	50.17±1.35^ab^	13.35±2.84^ab^	0.28±0.09^ab^
B_3_	2.76±0.76^a^	42.30±1.33^ab^	85.23±11.40^a^	45.59±9.02^a^	48.77±2.89^b^	14.40±1.74^ab^	0.41±0.18^a^
B_4_	2.29±0.39^a^	42.83±2.63^ab^	69.78±14.96^ab^	43.44±7.18^a^	54.83±2.08^a^	16.72±2.88^a^	0.39±0.17^a^

**Note:** Different small letters indicate the significant differences among five levels of biogas slurry addition.

### Effects of biogas slurry on fatty acids in *C*. *oleifera* oil

Saturated fatty acids mainly constitute of palmitic acid and stearic acid, and accounted for about 10% of the fatty acid content of *C*. *oleifera* oil in the present study (Table 3 and S1 Table). The effect of biogas slurry application on stearic acid was close to the significance level (p = 0.07), while it did not affect the unsaturated fatty acid content of fruit (Table 3 and S3 Table). Correlations among saturated and unsaturated fatty acids showed that oleic acid was negatively correlated with palmitic and linoleic acids, while linoleic acid was positively correlated with α-linolenic acid (Table 4 and S4 Table).

**Table 3:** Saturated and unsaturated fatty acids in the fruit of *C*. *oleifera* fertilized by different amounts of biogas slurry.

Treatments	Saturated fatty acid %		Unsaturated fatty acid %			
	Palmitic acid (C16:0)	Stearic acid (C18:0)	Oleic acid (C18:1)	Linoleic acid (C18:2)	γ- linolenic acid (C18:3)	α-linolenic acid (C18:3)
B_0_	8.713±0.235^a^	2.127±0.170^a^	79.743±1.25^a^	7.416±1.170^a^	0.005±0.001^a^	0.277±0.007^ab^
B_1_	7.925±0.337^b^	1.927±0.152^ab^	78.695±0.574^a^	7.804±0.485^a^	0.007±0.001^a^	0.252±0.017^b^
B_2_	7.927±0.427^b^	2.034±0.057^ab^	79.620±0.984^a^	7.286±0.284^a^	0.005±0.002^a^	0.260±0.013^ab^
B_3_	8.009±0.436^b^	1.820±0.059^b^	79.674±1.629^a^	7.695±1.308^a^	0.004±0.002^a^	0.301±0.033^a^
B_4_	7.982±0.138^b^	2.032±0.277^ab^	80.616±0.485^a^	6.533±0.511^a^	0.005±0.000^a^	0.241±0.031^b^

Note: Different small letters indicate the significant differences among five levels of biogas slurry addition.

**Table 4:** Correlations among saturated and unsaturated fatty acids.

Fatty acid	Correlation (significance: p-value)					
	Palmitic acid (C16:0)	Stearic acid (C18:0)	Oleic acid (C18:1)	Linoleic acid (C18:2)	γ- linolenic acid (C18:3)	α-linolenic acid(C18:3)
Palmitic acid (c16:0)	1					
Stearic acid (C18:0)	-0.359 (0.189)	1				
Oleic acid (C18:1)	-0.628* (0.012)	0.273 (0.325)	1			
Linoleic acid (C18:2)	0.441 (0.099)	-0.271 (0.328)	-.0924** (0.000)	1		
γ- linolenic acid (C18:3)	0.192 (0.493)	-0.261 (0.347)	-0.211 (0.451)	0.083 (0.769)	1	
α-linolenic acid (C18:3)	-0.174 (0.535)	-0.132 (0.639)	-0.375 (0.168)	0.567* (0.027)	0.036 (0.898)	1

Note

*indicates p<0.05, ≥0.01

**indicates p<0.01.

### Correlations of soil nutrients and fruit economic traits

The oil rate of fresh fruit was positively correlated with soil nutrients in 2015 (N, P, and K) and 2016 (organic matter; Table 5 and S5 Table). Oil rates of kernels were positively correlated with soil contents of N, P, K, and organic matter in both 2015 and 2016 (Table 5). Furthermore, oil yield was positively correlated with the concentrations of available P in both 2015 and 2016, and positively correlated with organic matter in 2015.

**Table 5:** Correlations of oil yield components and soil nutrients.

Nutrients of soil	Year	Correlation (significance: p-value)		
		Oil rate of fresh fruit	Oil rate of kernel	Oil yield
Available nitrogen	2015	0.241 (0.387)	0.549* (0.034)	0.407 (0.132)
	2016	0.514* (0.050)	0.720** (0.002)	0.418 (0.121)
Available phosphorus	2015	0.472 (0.076)	0.628* (0.012)	0.636* (0.011)
	2016	0.623* (0.013)	0.681** (0.005)	0.719** (0.003)
Available potassium	2015	0.454 (0.089)	0.648** (0.009)	0.252 (0.364)
	2016	0.591* (0.020)	0.647** (0.009)	0.392 (0.148)
Organic matter	2015	0.681** (0.005)	0.619* (0.014)	0.644* (0.010)
	2016	0.372 (0.172)	0.565* (0.028)	0.385 (0.157)

Note

*indicates p<0.05, ≥0.01

**indicates p<0.01.

## Discussion

*C*. *oleifera* is a woody tree species that is endemic to China, and an important economic plant. Camellia oil, the product of C. *oleifera*, is known to benefit health and is commonly used as food in China. Because flowers and fruits of *C*. *oleifera* grow throughout the year, and mature at the same time, fertilizers must be added to increase yields, especially of fruit and oil [30]. It has been reported that P, N, K, Ca, and Mg are the primary soil nutrients that limit the yield of *C*. *oleifera* [31]. In conventional planting, chemical fertilizers are widely used, which could result in land retirement, nutrient deficits, and the sealing of soil, and thus led to a reduction of yield [32]. To address this problem, this study used biogas slurry as alternative fertilizer, in *C*. *oleifera* plantation, and investigated the response of nutrients in rhizosphere soil as well as the yields of *C*. *oleifera*.

Biogas slurry is a secondary product of anaerobic digestion of bio-materials, and plays a central role in the efforts to improve the utilization of animal manure and to reduce the influence of animal excretion on surrounding environments [33, 34]. During manure digestion, about half of the carbon is released as methane and carbon dioxide (biogas), and part of the organic nitrogen is released as ammonium [35]. When it is applied to fields, ammonium can directly be utilized by crops. Furthermore, biogas slurry contains abundant available N, P and K, which are important nutrients for plants. It has been reported that the supply of N from digested slurry exerts a direct influence on the yield during the growing season, while the supply of P and K can be measured in the next year or the next several years [36]. Therefore, this study used a two-year experimental period to investigate the effects of biogas slurry on available N, P, and K of soils and the resulting yield of *C*. *oleifera*. During the two-year observation, fertilization with biogas slurry had positive effects on the increment of available N, P, and K of soils, and also improved the fruit and oil yields of *C*. *oleifera*. The results of this study indicated biogas slurry as an effective substitute for chemical fertilization in *C*. *oleifera* production.

Biogas slurry has an abundance of mineral elements and organic matter that are slowly released. These characteristics of biogas slurry may positively affect soil fertility indices, e.g., organic matter, available N, P, and K over many years [37]. A previous study evaluated the utilization ratio of NH_4_-N in biogas slurry, and reported that more than 90% of the applied NH_4_-N could be used, which indicated an immediate increase in the amount of soil NH_4_-N [9]. Friedel *et al*. reported a 37% increase in inorganic N during the incubation of farmyard manure-derived biogas slurry in soil for 60 days [38]. Similarly, this observed a sharp enhancement of available N in soils fertilized with as little as 10 kg of biogas slurry per plant in 2015. It has been speculated that the amounts of N supplied by biogas slurry in this study exceeded the demand of *C*. *oleifera*, therefore, available N accumulation was observed in 2016. The positive effects of available P and K after biogas slurry application were in accordance with that of available N. Available P is one of the main ecological factors that limits the increase of *C*. *oleifera* yield. Yuan *et al*. investigated the response of *C*. *oleifera* yield to low P and reported that *C*. *oleifera* roots secreted organic acids in response to low soil P, which led to the utilization of soluble phosphates [39]. This study only found a slight but not sharp decrease of soil available P in the control group in 2016. Kashem *et al* [40] demonstrated that an alkaline environment could promote the availability of P in soils. It is likely that alkaline biogas slurry in turns facilitates the absorption of P in soils. The slow release of nutrients in biogas slurry could contribute to the accumulation of organic matter, available N, P, and K during the second experimental year. The study predicts a larger promotion of nutrients in rhizosphere soil and yield of *C*. *oleifera* in response to long-term biogas slurry application.

## Conclusions

In the present study, the effects of biogas slurry on the nutrients in rhizosphere soil and fruit economic traits of *C*. *oleifera* have been investigated. Fertilization with biogas slurry significantly enhanced the concentration of available N, P, and K in soils, and significantly improved the yield of *C*. *oleifera*. During the first year, soils had higher concentrations of N, P, and K after application of biogas slurry and the promotion further continued during the second year. The yield of *C*. *oleifera* oil also increased over both experimental years, and if more biogas slurry was used, the yield was increased. The yield of oil also showed a correlation to the promotion of soil available N, P, and K in rhizosphere soils. Fertilization with30 kg/plant/year above (i.e., treatments B_3_ and B_4_) had the highest fresh fruit yield, fresh seed rate, and dry seed rate, and resulted in a higher oil yield per plant. Therefore, biogas slurry plays an important role in the production increase of *C*. *oleifera*, and might be an effective substitution for chemical fertilization in *C*. *oleifera* production.

## Supporting information

S1 Table Corresponding raw data of Fig 1. (XLSX)

S2 Table Corresponding raw data of Table 2. (XLSX)

S3 Table Corresponding raw data of Table 3. (XLSX)

S4 Table Corresponding raw data of Table 4. (XLSX)

S5 Table Corresponding raw data of Table 5. (XLSX)
